# 2416. Outcomes of Patients with Ventilator-Associated Pneumonia in For-Profit Hospitals Compared to Non-Profit hospitals: A National Retrospective Cohort

**DOI:** 10.1093/ofid/ofad500.2036

**Published:** 2023-11-27

**Authors:** Renato Bobadilla Leon, Garry Francis Morel

**Affiliations:** Guthrie Robert Packer Hospital, Sayre, Pennsylvania; Saint Barnabas Hospital Health System, Bronx, New York

## Abstract

**Background:**

Healthcare-associated infections (HAI) constitute a significant burden to patient outcomes and healthcare costs. Ventilator-associated pneumonia (VAP) carries a high mortality and represents one of the costliest HAI. There is uncertainty if treating this subgroup of patients in For-Profit (FP) hospitals portents better outcomes than Nonprofit (NP) hospitals.

**Methods:**

A retrospective cohort of patients admitted from January 2016 through December 2020 was extracted from the National Inpatient Sample database. We compared two cohorts of patients with a primary diagnosis of VAP: patients admitted to FP hospitals and patients admitted to NP hospitals. The primary end-point was all-cause inpatient mortality. A multivariate logistic regression analysis was conducted. The secondary end-points included: comparing the requirement of vasopressor use and Acute Kidney Injury (AKI) requiring hemodialysis (HD); describing associated comorbidities; and comparing if there was a difference in length of stay (LoS) and hospital cost between the 2 groups. In order to compare LoS and hospital cost, a multivariate linear regression was conducted.

**Results:**

A total of 6 155 hospitalizations under the primary diagnosis of VAP were included. No statistically significant difference in mortality was noted (FP: 4.24 vs. NP:8; p = 0.182), but age (aOR 1.01, p = 0.020) and Charlson Comorbidity Index(CCI) (aOR 1.20; p < 0.001) were modest predictors of mortality for both groups (FP vs. NP). There was no statistically significant difference in mean LoS between these two groups (FP:10.61 days vs. NP: 9.57, p=0.398). CCI (Coef 0.64, p= 0.01) and admission to large-size hospitals (Coef 3.03, p < 0.001) were predictors of LoS for both groups. Admission to for-profit hospitals predicted higher hospitalization costs (p = 0.020). Furthermore, admission to medium-size hospitals, large-size hospitals, and urban hospitals were also predictors of higher hospital cost (p < 0.05). No statistically significant difference was found in vasopressor use and AKI requiring HD among both groups (FP vs. NP) (p < 0.05).

**Conclusion:**

Admission to For-Profit hospitals did not portend better outcomes compared to Nonprofit hospitals. Conversely, admission to For-Profit hospitals predicted higher total hospital costs.

**Disclosures:**

**All Authors**: No reported disclosures

